# First Expert Evaluation of a New Steerable Catheter in an Isolated Beating Heart

**DOI:** 10.1007/s13239-020-00499-3

**Published:** 2020-11-18

**Authors:** Awaz Ali, Tamas Szili-Torok, Marco Stijnen, Paul Breedveld, Dimitra Dodou

**Affiliations:** 1 BioMechanical Engineering, Delft University of Technology, Delft, Zuid-Holland The Netherlands; 2 Erasmus Medical Center, Electrophysiology, Rotterdam, Zuid-Holland The Netherlands; 3 LifeTec Group, Eindhoven, The Netherlands

**Keywords:** Expert opinion, Evaluation, Steerable catheter, Beating heart, Catheter design

## Abstract

**Purpose:**

In previous studies we developed two mechanical prototypes of steerable catheters: the *Sigma*, which uses joysticks to actuate two steerable tip segments, and the *Epsilon*, which has a handle that is an enlarged version of the tip. In this study, we present a first performance evaluation of the prototypes in the cardiac environment. The evaluation was carried out by an expert user, an electrophysiologist with over 20 years of experience, to obtain insight in clinically relevant factors.

**Methods:**

Two experiments were conducted. In the first experiment, the *Sigma* was used in a passive beating heart setup connected to pumps with a saline solution and camera visualization, and compared with the expert’s past experience with conventional steerable catheters. In the second experiment, the *Sigma* was used in an active beating heart setup with blood perfusion through the coronary arteries and echo visualization, and compared with the *Epsilon* prototype. The prototype was evaluated through questionnaires on task performance, catheter usability, and workload. After each of the experiments, the catheter characteristics were evaluated *via* a survey and followed by an in-depth interview.

**Results & Conclusions:**

The expert user found the passive beating heart setup to more successful than the active beating heart setup for the purpose of this experiment, with insightful visualization while the heart was in beating condition. The steerability of the prototypes was experienced as useful and clinically relevant. Based on the questionnaires and interview we were able to identify future design improvements and developments for the steerable catheter prototypes.

**Electronic supplementary material:**

The online version of this article (10.1007/s13239-020-00499-3) contains supplementary material, which is available to authorized users.

## Introduction

Catheters and sheaths are used to treat and diagnose disorders in the majority of cardiac interventions. Interventions include, for example, radiofrequency ablation to treat cardiac arrhythmia, replacement of calcified or leaking heart valves, and obtaining samples from the heart tissue during endo-mycoardial biopsy. Precise manipulation of cardiac catheters remains a complicated task, due to the dynamic cardiac environment and the limited freedom of catheter movement.^5^,^13^ Mal-positioning the catheter tip increases the risk for multiple intra- and post-operative complications, including vascular and cardiac damage, cardiac perforation, and thromboembolic events.^6^,^16^,^20^

**Figure 1:**
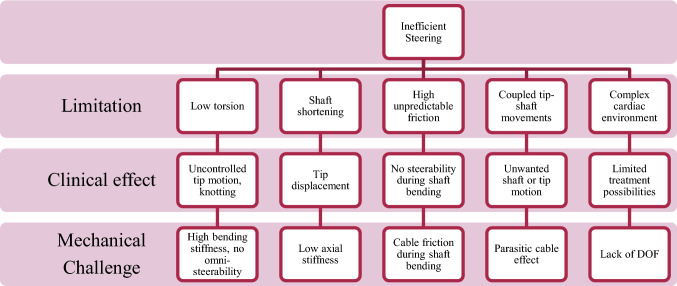
Overview of limitations, clinical effects, and mechanical challenges that were identified and solved in previous research. Figure adopted and modified from Ref. 2.

Over the past few years, different types of steerable cardiac catheters have been presented in literature.^1^,^8^,^17^,^21^ Similarly, a number of different steerable catheter systems have become available commercially in recent years. Besides the regular push/pull and torque movements, steerable catheters may have additional Degrees of Freedom (DOF) and one or more deflectable segments in the tip that allow the interventionist to navigate through the cardiac environment and precisely position the tip. As of today, commercially available steerable systems include the manually steerable introducer sheath AgilisTM NxT (St. Jude Medical, St. Paul, MN, USA), the robotic catheter systems Sensei^®^ X and MagellanTM (Hansen Medical, Mountain View, CA, USA), and the remote magnetic catheter navigation system Niobe (Stereotaxis, St. Louis, MO, USA). Clinical trials have shown significant improvements in procedural time and patient safety in interventional cardiology and electrophysiology interventions when using steerable catheters, as compared to non-steerable ones.^7^,^14^,^15^,^19^ In some cases, the use of steerable catheters allowed for successful treatment of cardiac patients whom could not be treated *via* any other trans-catheter approach.^11^,^15^,^18^ While clinical results have been promising, the current steering potential of mechanically steerable catheters is limited due to a number of underlying mechanical challenges. This includes high bending stiffness, low axial stiffness, friction between inner components, and parasitic cable effects, see Fig. 1.

**Figure 2:**
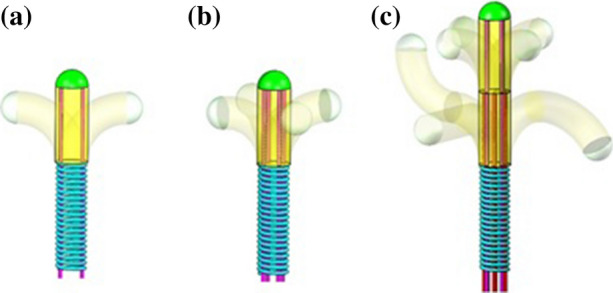
The concept of catheter steering with multiple segments explained in DOF, with (a) 1 DOF steering with one steerable segment, (b) 2 DOF steering with one steerable segment, and c) 4 DOF steering with two steerable segments. Image adopted from Ref. 2.

In addition to the aforementioned challenges, the majority of steerable catheters also have 1 Degree of Freedom (DOF),^1^ and the manoeuvring of the instrument tip is therefore limited to single planar movements. To improve catheter steering by solving some of the aforementioned challenges, we previously developed a multi-steerable catheter, the *Sigma*.^2^ The *Sigma* catheter is a 4-DOF instrument that steers over multiple planes simultaneously by using two steerable segments, see Fig. 2. The catheter is able to create S-shaped curves and move along circular pathways using solely mechanical actuation. The two tip segments are actuated by two independent joysticks operated by the thumb and index fingers of the same hand. An internal friction mechanism locks each of the joysticks in position upon releasing the joystick. As such, the tip segments remain in the designated curve until the joystick position is changed. The *Sigma* has a 1-m long shaft with a total outer diameter of 3 mm and a lumen of 1.5 mm.

**Figure 3:**
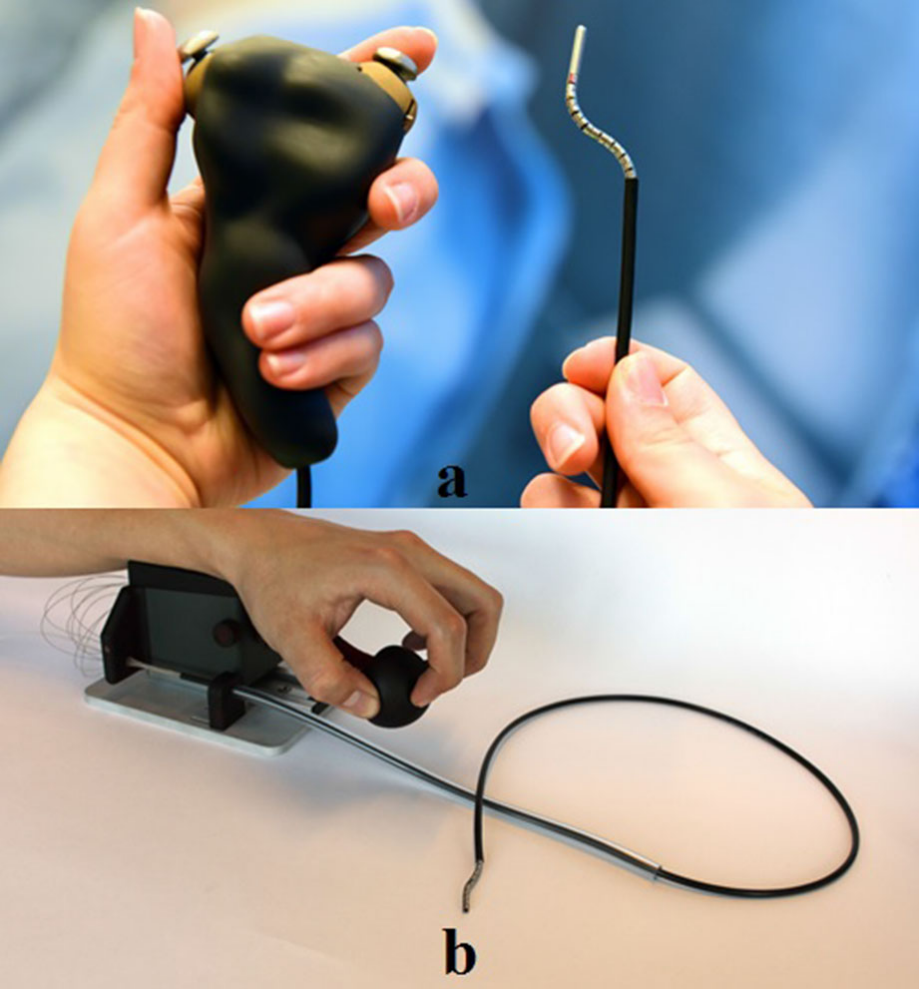
Prototypes of (a) the Sigma catheter with joystick control through thumb and index finger and (b) the Epsilon catheter with a handle that is an enlarged version of the tip.

To improve and simplify control of the *Sigma*, in follow-up research we designed a second catheter, the *Epsilon*. The *Epsilon* has a simplified control because instead of two joysticks, it has a handle that is an enlarged version of the tip. As such, it allows the operator to manually form the shape of the handle, which is copied by the catheter tip. Additionally, the *Epsilon* catheter allows for longitudinal motion by pushing the whole construction forward from the pols, thereby freeing the other hand and allowing a fifth DOF. Prototypes of both catheters are shown in Fig. 3.

The *Sigma* and *Epsilon* catheter prototypes were previously validated in technical feasibility and user tests. In the latter, both catheters were tested by five novice users who were asked to reach various targets in an experimental setup. In total, 45 tasks were carried out and results showed that the highest level of steerability (4 DOF) allows more complex pathways/tasks to be completed but that average completion time takes longer. Even though these tests provided insight regarding the functionality and steerability of the new catheters along complex pathways, it remains unknown how the catheters perform in the natural dynamic cardiovascular environment. In this paper, we aimed to investigate the performance of the prototypes in the cardiac environment and under limited instrument visibility. Accordingly, two experiments were conducted: one with a passive beating heart setup connected to pumps with a saline solution and camera visualization and a second experiment with an active beating heart setup with natural blood perfusion through the coronary arteries and with echo visualization. The evaluation was carried out by an expert user, to obtain insight in clinically relevant factors.

## Methods

### Beating Heart Setup

To test the functionality of the catheter in the cardiac environment, the *Sigma* catheter was used in both the passive and active beating heart setup. Both setups were developed by LifeTec Group (LifeTec Group BV, Eindhoven, The Netherlands). All hearts used in this study (*N* = 2, 1 for each experiment) were obtained from pigs that were slaughtered for human consumption and were retrieved according to standard protocols for usage of porcine tissue intended for human consumption.^3^ The protocols at the slaughterhouse and laboratory were developed in accordance with EC regulations 1774/2002 regarding the use of slaughterhouse animal material for diagnosis and research, supervised by the Dutch Government (Dutch Ministry of Agriculture, Nature and Food Quality) and approved by the associated legal authorities of animal welfare (Food and Consumer Product Safety Authority). These protocols prevent additional animal suffering and sacrifice in performing the experiments.

**Figure 4:**
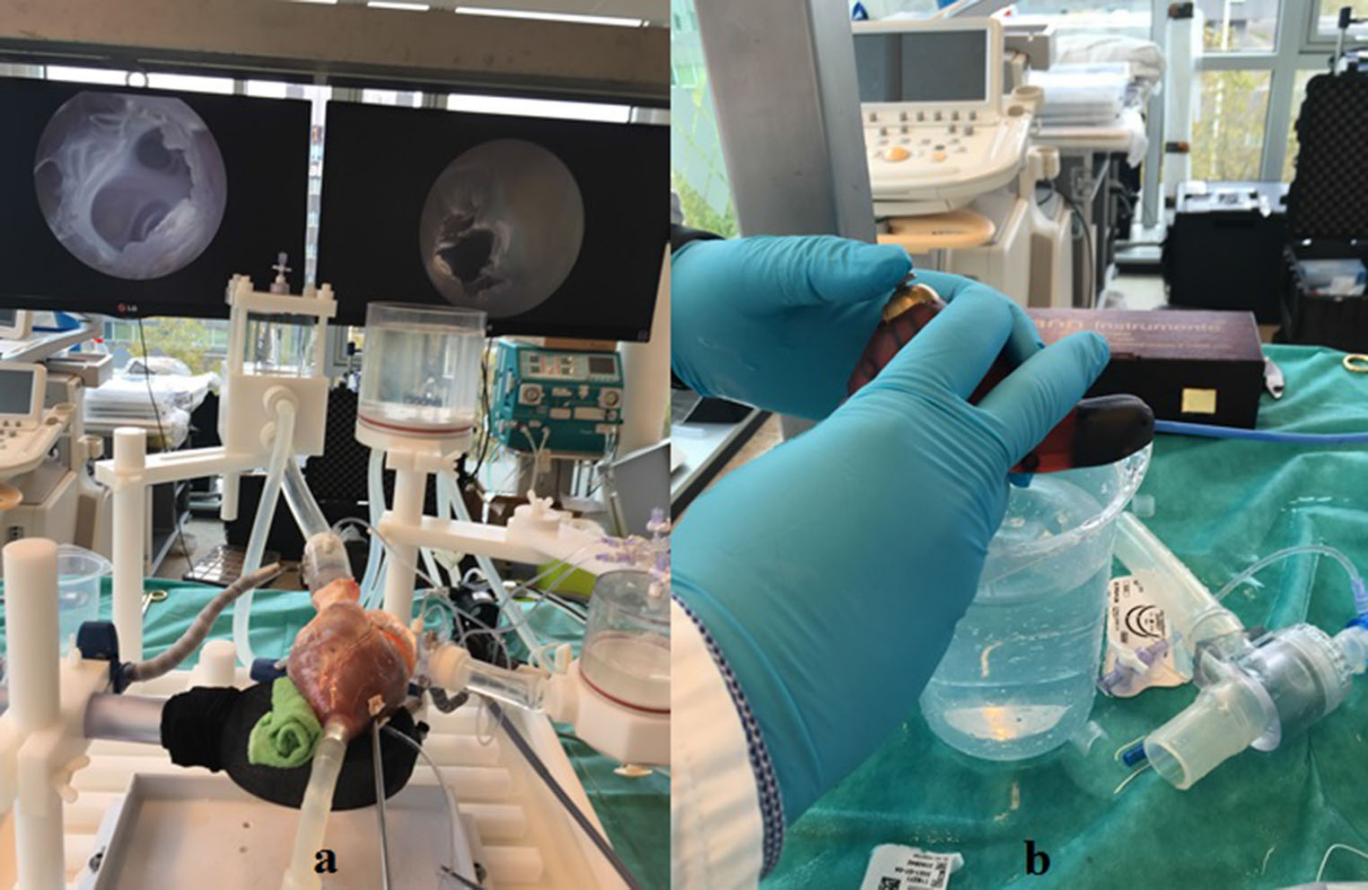
Preparation of the passive beating heart setup: (a) passive beating heart prepared by connection of a pulsatile dynamic fluid system with a saline solution to a porcine heart (b) handle of the steerable *Sigma* catheter used in the experiment.

The passive beating heart setup, or cardiac bio-simulator, is an *in vitro* platform that simulates the beating heart motion through a connection with pumps with a saline solution running through. The setup models heart valve and hemodynamic function in a heart and has proven to be a useful tool for preclinical training in earlier studies. The overall setup is described by Leopaldi *et al*.^12^ and consists of a pulsatile dynamic fluid system that is connected to a porcine heart. The left ventricle of the heart is exposed to a cyclic pressure to recreate physiological beating heart movement using a pulse duplicator system that is attached *via* a cannula in the apex. The setup is combined with intracardiac videoscopic visualization through a transesophageal echo probe in the left atrium and as such, a saline solution is used to replace blood while maintaining visibility. Using this setup, the cardiac chamber pressures are monitored and can be regulated to recreate the required *in vivo* physiological circumstances. Figure 4 shows a photograph of the setup.

**Figure 5:**
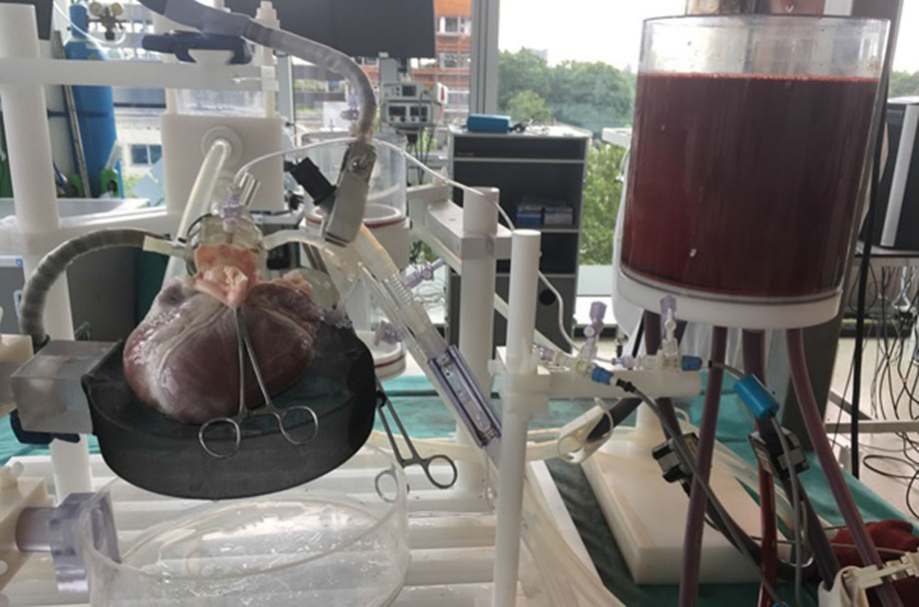
Preparation of the active beating heart setup by cannulating a porcine heart and mounting it in a circulation platform with porcine blood.

The active beating heart setup, or Physio-heart, was developed according to the procedure described by De Weger and De Hart.^3^,^4^ This setup models a normal working human heart with a cardiac output of 3.5 L/min, normal left ventricular and aortic pressures (120/80 mm Hg and heart rate 85 bpm), and physiological pressure and flow pulses. The porcine heart that was used in the setup was obtained from a slaughterhouse following a procedure similar to transplantation protocols^3^ and cooled on ice directly after harvesting. 6 L of fresh blood were obtained from the same source, heparinized, and kept cooled during transport. The heart was prepared and cannulated to be mounted in a circulation platform developed by LifeTec Group. A pre-existing tear in the right atrium (due to explantation in the slaughterhouse) was sutured to prevent leakage during the experiment. The heart was further prepared by removing the pulmonary veins at the left atrium and by sealing the veins. Next, a cannula was fixed to the aorta and another to the pulmonary artery, allowing for a connection to the external blood circulation. The hearth was revived by pumping warm and oxygenated blood (37.0 ± 0.5 °C) from a reservoir into the aortic root, inducing flow into the coronary arteries after which myocardial contractions were initiated. The heart was defibrillated to obtain synchronous contraction patterns and to stabilize the heart rhythm. During the experiment, hemodynamic parameters including heart rate and blood flow were monitored. A Philips iE33 echocardiography system was used for viewing purposes. Figure 5 shows an overview of the active beating heart setup.

### Experimental tasks

During the first experiment, the *Sigma* catheter was tested by the clinical expert in the passive beating heart setup. During the second experiment, the *Sigma* catheter was tested in the active beating heart setup. Each experiment started with a practice phase, during which the expert was instructed to manoeuvre the *Sigma* catheter in space to obtain familiarity with the steering mechanism. Following the practise phase, the LifeTec Group team inserted the catheter into the right side of the heart through the vena cava, right atrium, and through the atrial septum to enter the left atrium. Once the catheter had reached to left atrium, the trial phase started and the clinician was instructed to carry out positioning tasks at a number of different locations. The locations included the left and right pulmonary veins, the mitral valve, the valve annulus, the papillary muscles in the left ventricle, and the aortic valve. At the end of the first experiment, the clinician was instructed to compare the *Sigma* catheter to conventional catheters. At the end of the second experiment, the clinician was instructed to compare the *Sigma* catheter with the *Epsilon* catheter tip in free space. Free space was defined as space without matter, thus the catheter was placed on a table while the clinician was holding the shaft and manipulating the catheter tip in air without contacting any objects.

### Questionnaires

Prior to both experiments, the clinician was asked to fill in a survey focusing on basic demographic characteristics, his clinical expertise, types of difficult procedures and tasks he faces on a regular basis, and his experience with the use of steerable catheters or sheaths.

Following each experiment, the clinician was instructed to complete three questionnaires focusing on (1) task performance, (2) usability of the catheters, and (3) user workload. Each questionnaire was completed for: (1) the clinician’s previous experience with conventional steerable catheters, (2) the clinician’s experience with the *Sigma* catheter in the passive beating heart setup, (3) the clinician’s experience with the *Sigma* catheter in the active beating heart setup, and (4) the clinician’s experience with the *Epsilon* catheter in free space. The three questionnaires were printed on a size A3 paper, and each questionnaire was accompanied by an example question and answering method at the top of the page.

The task performance questionnaire asked the clinician to assess the ease, speed, and precision by which he could manoeuvre along the pathways, reach the endpoint, and steer in the direction of preference when using each of the catheters. Each combination (i.e., ease, speed, and precision of pathways, endpoint, and direction) was rated by the clinician on a 10-point scale, ranging between ‘very difficult’, ‘very slow’, or ‘very imprecise’ and ‘very easy’, ‘very fast’, or ‘very precise’.

The usability questionnaire was a standard System Usability Scale (SUS) in which the clinician had to score 10 items on a 5-point Likert scale with 1 being ‘strongly disagree’ and 5 being ‘strongly agree’.^10^ For each of the odd items (1, 3, 5, 7, 9) one point was subtracted from the user response, whereas for even-numbered items (2, 4, 6, 8, 10) the user response was subtracted from 5. This allowed all values to be scaled from 0 to 4 instead of from 1 to 5, with 4 being the most positive response. Subsequently, all converted responses were added up and multiplied by 25 to convert the range of possibilities from 0 to 100.^10^ SUS is frequently used in user experience experiments for newly developed products in various fields.

The workload was measured using the NASA Task Load Index (TLX) with the ‘Raw TLX’ score.^9^ TLX allows the user to rate the mental, physical, and temporal demand, as well as the performance, effort, and frustration on a 20-point scale. Each of the six TLX factors was rated with ticks ranging between 1 and 20. The number of each of the ticks was multiplied with 5 to obtain a value on a scale of 100.

At the end of each experiment and after answering the three task-related questionnaires, the clinician was instructed to rate the catheter characteristics through a survey. The survey consisted of two parts. In the first part, a number of characteristics of the used catheter, such as ‘level of steerability’, ‘level of tip control’, ‘maintaining a specific curve’, or ‘maintaining instrument-tip contact’, had to be compared to commercially available catheters using a 5-point Likert-scale from ‘significantly worse’ to ‘significantly better’. The second part of the survey asked how likely it was that a multi-steerable catheter could lead to ‘improved manoeuvrability’, ‘easier catheter sliding’, ‘shorter procedure time’, or ‘fewer common complications’ on a 5-point Likert-scale between ‘very unlikely’ and ‘very likely’.

Finally, an in-depth interview was carried out after each of the two experiments. In the first interview, the *Sigma* catheter and conventional steerable catheters were compared, whereas in the second interview, the *Sigma* and *Epsilon* catheters were compared. The question topics included the overall experience, the degree of steerability, the handle, and the future use of the instrument. Both interviews were audio-recorded with permission of the expert clinician.

**Figure 6:**
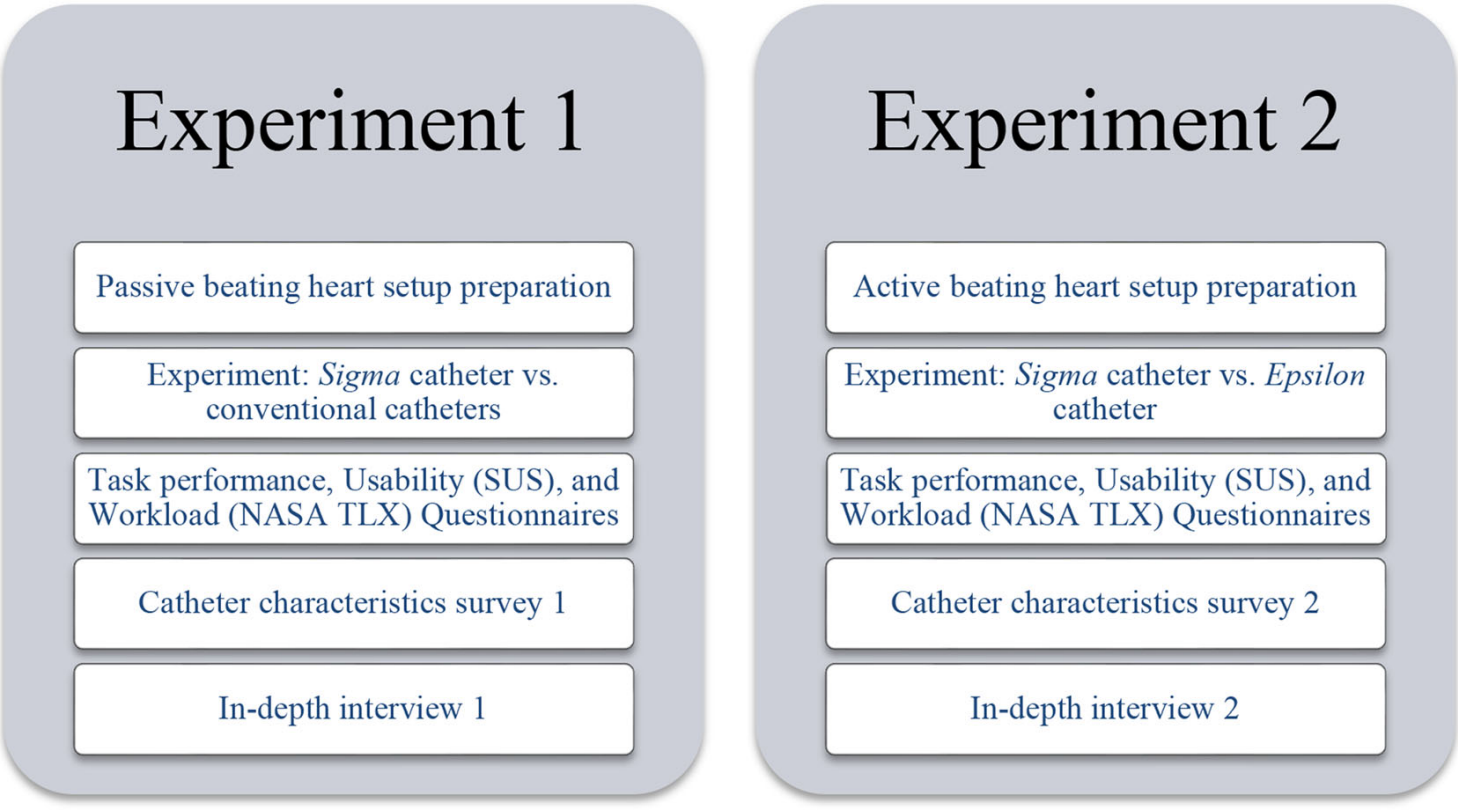
Flowchart representing both experiments.

Figure 6 shows an overview of the components in each of the experiments. All questionnaires and surveys are available in the supplementary materials.

## Results

### Expert’s background

The clinical expert (the second author) was a 48-year old male cardiologist, specialised in electrophysiology. His clinical training consisted of the cardiology specialisation, an electro-physiology fellowship, a PhD degree, and a clinical fellowship. The clinician had practised his specialism as an all-round electrophysiologist for over 20 years, carrying out electrophysiology procedures between 10 and 20 h per week. Prior to the experiment, he had experience with all available steerable sheaths and catheters during electrophysiology procedures and preferred to use steerable catheters over non-steerable ones during most procedures. On the question which cardiac pathway is the most complex to move a catheter through, he responded that there is no definite difference between pathways, because each of them can be challenging or rather easy, depending on the patient and the circumstances. On the question which cardiac location is the most complex to reach, he answered that the postero-septal mitral valve and right inferior pulmonary vein are the most complex.

### Passive and Active Beating Heart Experiments

#### Passive Beating Heart

Prior to starting the experiment with the passive beating heart, the catheter was placed in the vena cava, to the right atrium, and through the atrial septum. Using this postero-septal approach, the session started. The clinician was able to reach the mitral valve with the tip of the *Sigma* catheter and to move the tip along the valve annulus, thereby contacting multiple locations of the valve with the tip, including the posterior leaflet locations such as p1 and p2. Next, he was able to move the catheter tip to the pulmonary veins and reach the left superior and inferior veins. The right superior and inferior pulmonary veins could not be contacted, because the catheter tip was too long to manoeuvre freely inside the relatively small porcine atrium. During the next step, the catheter was retracted and placed in the aortic pathway. The clinician was able to move the catheter tip through the aortic valve and manoeuvre it through the left ventricle toward the mitral valve. There, once again, he was able to reach all valve structures of interest, including the papillary muscle, while also avoiding contact with the *chordae tendineae*.

#### Active Beating Heart

**Figure 7:**
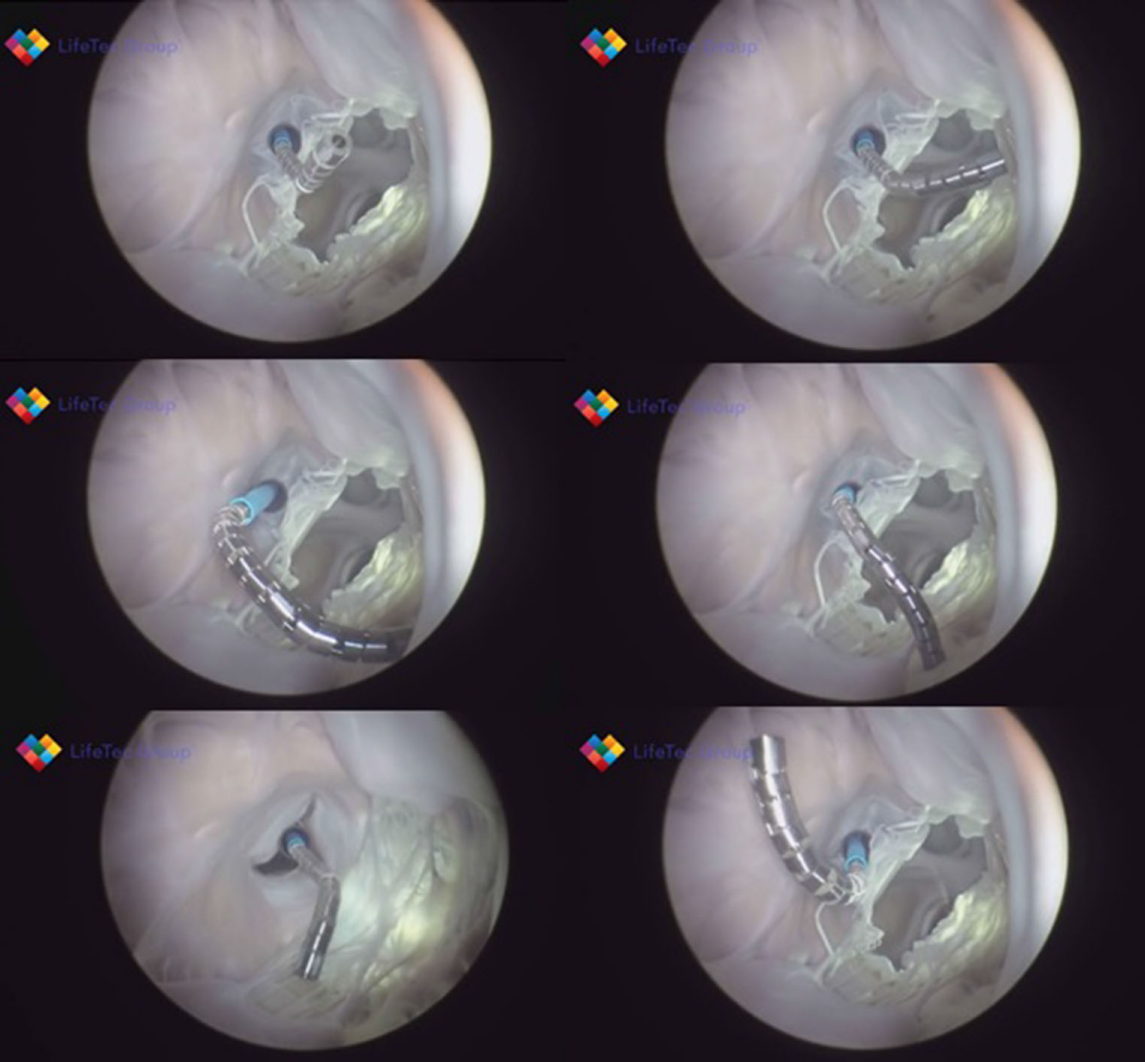
Endoscopic views of the *Sigma* catheter tip in the passive beating heart in various positions in the ventricle.

**Figure 8:**
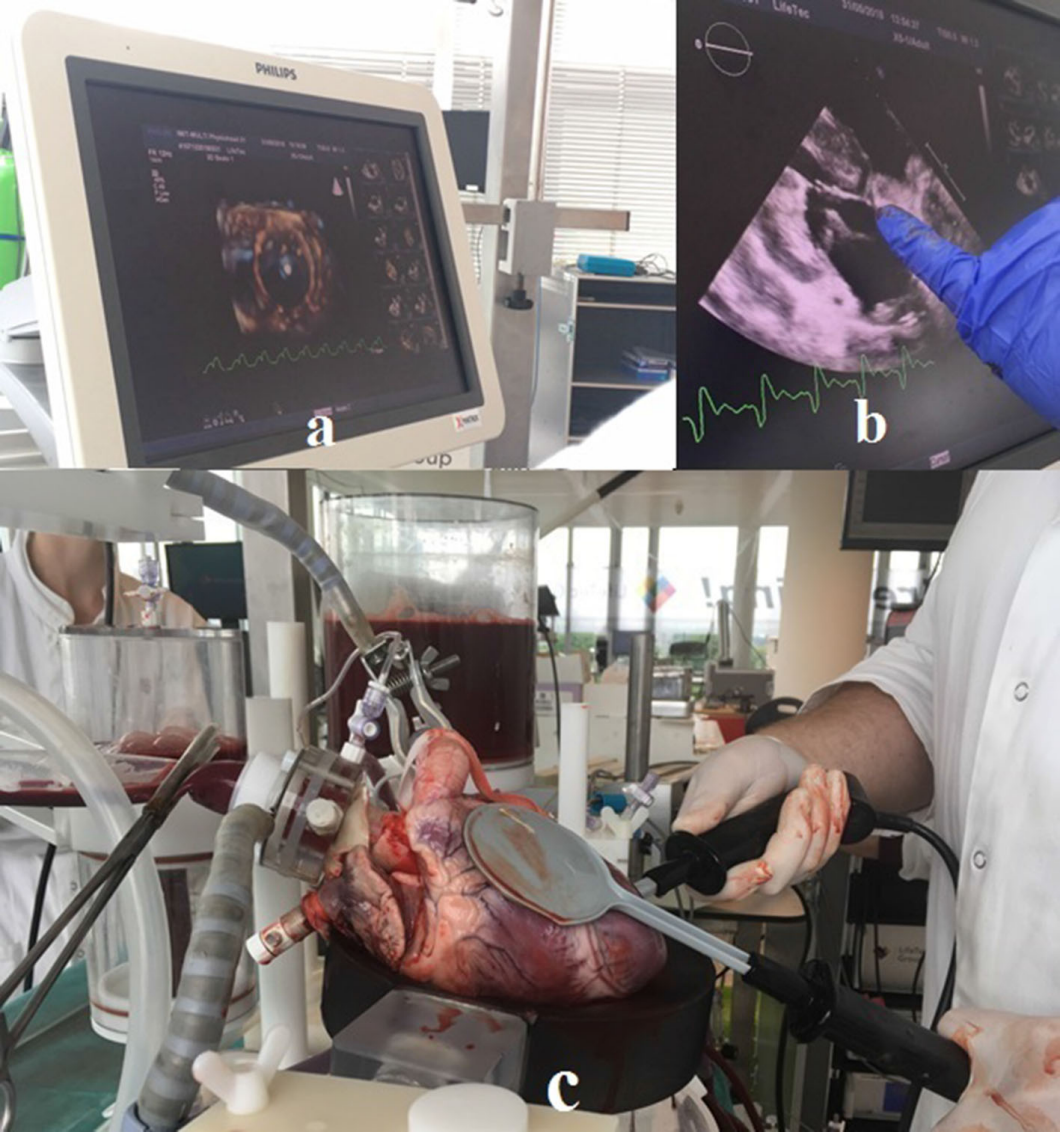
(a and b) Echocardiography views of the *Sigma* catheter tip in the active beating heart, (c) cardiac fibrillation to start the cardiac beating motion.

Prior to starting the session with the active beating heart, a small opening was made in the atrial septum to allow the catheter to go through without the use of a septal puncture needle and sheath. The catheter was placed inside the vena cava and from that location manipulated by the clinician to the right atrium to cross the atrial septum to the left atrium. The opening and the catheter could not be visualized clearly on the echo images. In addition, because there were no septal puncture sheath and needle available at the location, crossing the atrial septum with the catheter became an impossible instruction. After multiple attempts with the *Sigma* to reach the left side of the heart through the atrial septum, it was decided to create an apical opening for catheter placement. From the apical entrance, the clinician was able to steer the catheter to reach the right pulmonary veins and the papillary muscles. Additionally, he was able to turn the catheter tip 360° and create a circular pathway. Figures 7 and 8 show images of the catheter in the passive and the active beating heart setup.

### Task Performance

**Figure 9:**
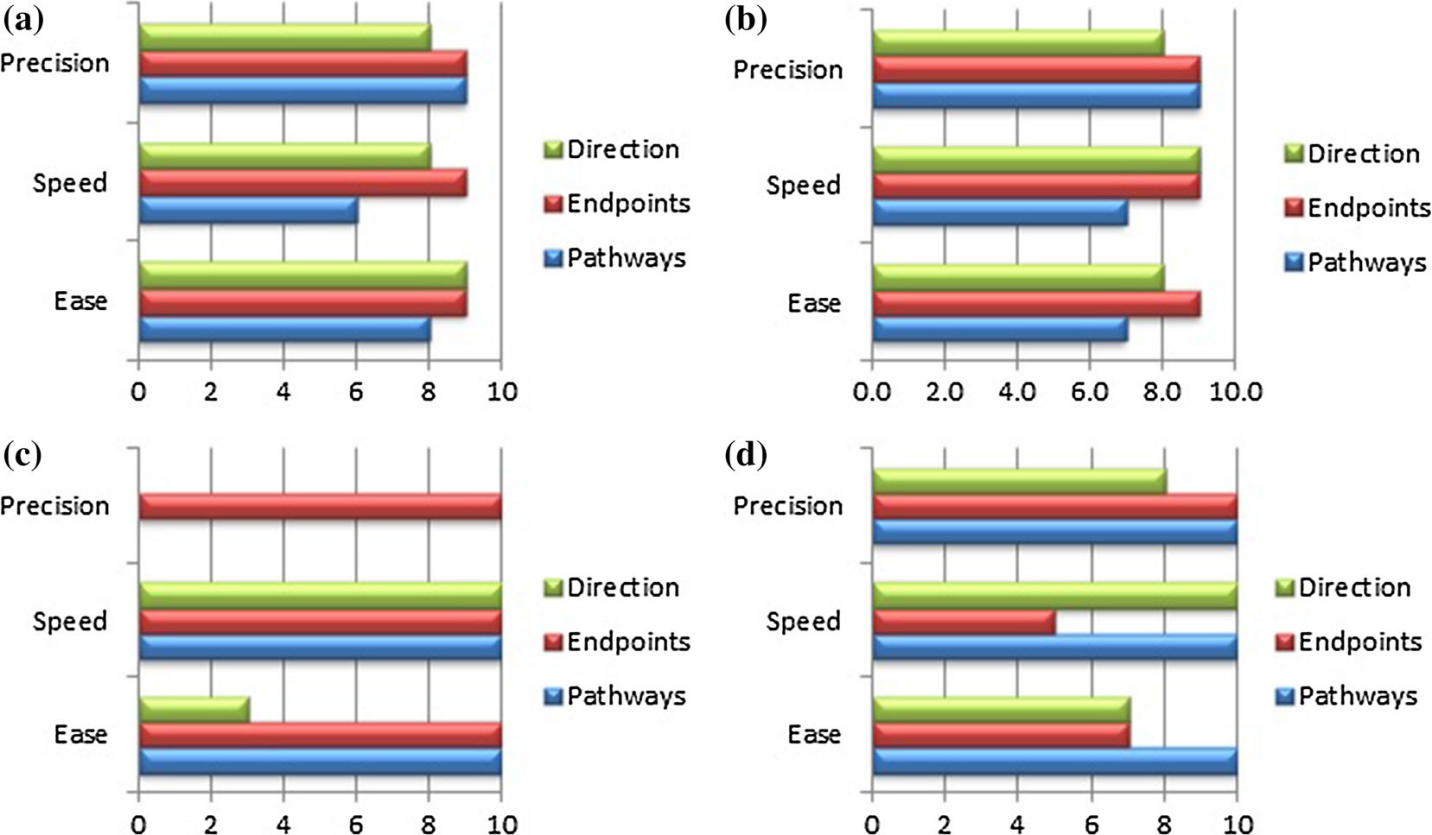
Task performance scores for (a) conventional steerable catheters from previous experience, (b) the *Sigma* catheter in the passive beating heart experiment, (c) the *Sigma* catheter in the active beating heart experiment, and (d) the *Epsilon* catheter in air.

Figure 9 shows the task performance evaluation of steerable catheters based on previous experience and of the two prototypes based on the experimental evaluation. The *Sigma* catheter in the active beating heart setup scored the highest on precision (10) followed by the *Epsilon* catheter tested in air (9.3). The Sigma catheter in the passive beating heart setup and the conventional steerable catheters equally scored 8.7. For speed, the *Sigma* catheter in the active beating heart scored the highest (10) followed by the *Sigma* catheter in the passive beating heart (8.3) and the *Epsilon* catheter in air (8.3). For ease of use, conventional catheters scored the highest (8.7), followed by the *Sigma* catheter in the passive beating heart setup (8), and the *Epsilon* catheter (8). Here, the *Sigma* catheter in the active beating heart setup scored the lowest (7.7.) due to a score of 3 on ease of use in choosing the steering direction. Precision in manoeuvring along the pathways and steering in the direction of preference were not scored for the Sigma catheter in the active beating heart experiment (Fig. 7c), because the clinician had indicated he was not able to test both of these factors under the presented circumstances.

### Usability

The clinician scored the *Sigma* catheter in the passive beating heart experiment, the *Sigma* catheter in the active beating heart experiment, and the *Epsilon* catheter in air with a score of 100/100 based on the 10 items. Conventional catheters scored 86/100, with the speed of learning being rated with the lowest score.

### Workload

**Figure 10:**
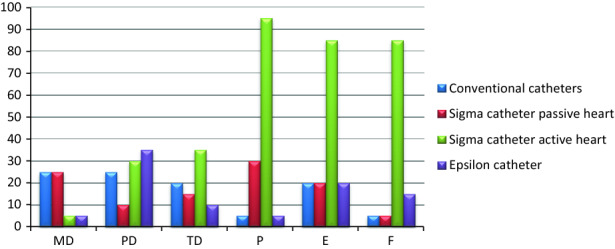
Overview of NASA TLX raw data for each of the catheters. *MD* mental demand, *PD* physical demand, *TD* temporal demand, *P* performance, *E* effort, *F* frustration.

Figure 10 shows the workload evaluation of the prototypes and the conventional catheters. Mental demand was scored the lowest (5) for the *Sigma* catheter in the active beating heart experiment and the *Epsilon* catheter. Physical demand was scored the lowest (10) for the *Sigma* catheter in the passive beating heart. For temporal demand, the *Epsilon* catheter was rated the lowest (10). For performance, conventional catheters and the *Epsilon* catheter were rated the lowest, thus having the performance closest to perfect (5). The *Sigma* catheter in the active beating heart scored considerably high in terms of effort (85) and frustration (85).

### Catheter Characteristics

**Figure 11:**
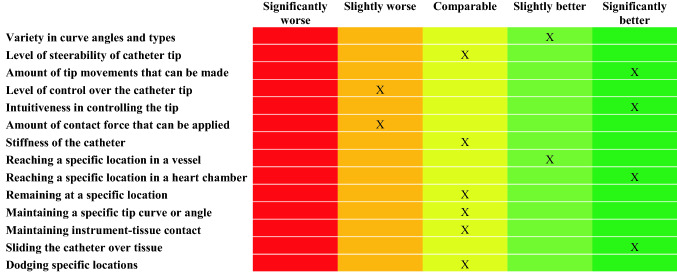
Catheter characteristics of the *Sigma* compared to conventional catheters.

Figure 11 shows the evaluation of the catheter characteristics. The *Sigma* catheter was rated as ‘significantly better’ than conventional steerable catheters in the amount of movements that could be made with the tip, the intuitiveness in controlling the tip, reaching a specific location in a heart chamber, and sliding the catheter over tissue. The level of control over the catheter tip and the amount of contact force that could be applied were the only two characteristics on which the *Sigma* was rated ‘slightly worse’ than conventional steerable catheters.

The clinician was asked to complete the same survey to compare the actuation method of the *Epsilon* catheter with that of the *Sigma* catheter. The *Epsilon* catheter was rated as ‘significantly better’ than the *Sigma* in terms of variety of curve angles, curve types, and the amount of movements that could be made with the catheter tip. The intuitiveness in controlling the tip was rated as ‘slightly better’. All other characteristics were rated as comparable between the two catheters.

### Prediction of Effects

**Figure 12:**
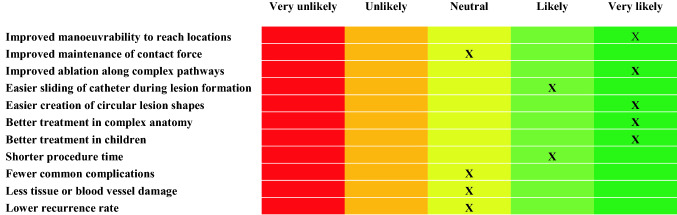
Prediction of effects for the *Sigma* catheter in comparison with conventional catheters.

Figure 12 shows an overview of the prediction of effects. All investigated effects were rated as ‘very likely’ or ‘likely’ to occur using the *Sigma* catheter, except for improved maintenance of appropriate contact force, fewer common complications, less tissue or blood vessel damage, and lower recurrence rate, for which the prediction was rated as neutral.

The clinician completed the same survey for the *Epsilon* catheter in comparison with the *Sigma* catheter. Here, the effects that were rated as ‘very likely’ to occur using the *Epsilon* catheter were improved manoeuvrability, easier creation of circular lesions, and better treatment in complex anatomy. Shorter procedure time was rated as ‘likely’. All other effects were rated as neutral when comparing the *Epsilon* with the *Sigma* catheter.

### Interview

#### General Experience

The clinician experienced the experiment as realistic. Compared to a clinical situation, he experienced the visual feedback that was generated in the passive beating heart setup with endoscopic cameras as very insightful. In the active beating heart setup with only echo available for visualisation, it was difficult to see the catheter and to judge its overall performance. The clinician argued that, during a real procedure, the visualisation offers more possibilities in terms of localizing the catheter tip and judging the catheter performance.

The clinician stated that, with the *Sigma* catheter in the passive beating heart, the maximum achievable curve was too small and that the catheter should have a maximum curve of 270° to reach all structures. The clinician found the *Sigma* more intuitive and easier to learn than conventional catheters, mostly because of the handle. With conventional catheters, he argued, time is needed to learn and understand the working principle, especially when catheters have asymmetric curves, where activating either one of the small or one of the large curves requires trial and error. He found that the *Sigma* allowed him to work as he prefers to do in the clinical situation: having a target and steering the tip around it. He argued that the level of tip softness makes it safer to use, such as during pulmonary vein isolation, but that this softness also makes it difficult to insert the catheter inside the body initially. Comparing the *Sigma* with the improved *Epsilon* catheter, he found the *Epsilon* to be more intuitive, because the tip precisely followed the handle. However, as a disadvantage, he found that the *Epsilon* handle required more and larger hand movements to reach the same level of steerability as the *Sigma* catheter.

#### Tip Steerability

The clinician’s experience in using the multi-steerable *Sigma* catheter was generally positive. He indicated that, at almost all locations inside the heart, it was possible to separately move the two segments, except for when one of the segments was too close to the cardiac wall, in which case the proximal segment was not pushing away the tissue but was confined by it. He found it difficult to judge whether this extreme flexibility would be clinically useful. When asked to compare the manoeuvrability and steerability of the multi-steerable catheter with commercially available ones, he answered: “*The Sigma is much faster. When you move the tip it has a one*-*to*-*one reaction, which is very good, but this is only good when you have relatively limited force at the tip and this one has it. There are different steerable sheaths on the market, some with a turning knob, and that by nature is very slow. Others, such as bidirectional ones with push*–*pull mechanisms, could be faster too, but they could also be dangerous because they are very stiff. The multi*-*steerable catheter is much faster and smoother, and there I see the major potential. And it’s not a small difference, it’s a very big difference in the speed and the reaction time.*”

The clinician found that *Sigma* was extremely reliable when moved in air, very reliable at the beginning of the experiment after having inserted the catheter in the heart, and less reliable after a while, when the catheter was inside the heart. He attributed this deterioration of reliability over time to the joysticks becoming rough to move due to blood entering them. He found the *Sigma* easy to work with in reaching difficult locations, for example under the valves. He was able to make all desired curves under the mitral valve and stated that such curves normally take fellows a year to learn with conventional catheters. Comparing the *Sigma* and *Epsilon* catheters, he argued that the *Epsilon* catheter would perform better inside the heart, mainly because the proximal tip of the *Epsilon* was better to manipulate.

#### Handle Actuation

In using the joysticks of the handle to steer with, the clinician stated the following: “*The joysticks are fantastic. It is not comparable to the ones we have, it is much better. Except for the fact that the shaft should come out of the front of the handle and not out of the back forming a U*-*shape. Otherwise in some patients you will not be able to turn the handle while the shaft is placed in a U*-*turn.*” For the response speed of the tip to the joystick movements he stated: “*It was not only relatively fast, but very fast, close to perfect. I would say one*-*to*-*one reaction. Many times there is a delay in our current catheters, but with this one I didn’t feel a delay.*” He found that he did not have to look back at the joysticks during the procedure to see what he was doing, but that when he got stuck with the tip somewhere, it was useful to look back at the handle and place both joysticks in the normal position to restart from a neutral position again. Additionally, the clinician appreciated that the tip remained in the chosen curve once the joystick had been actuated. One improvement for the handle would be to add a clicking mechanism, to indicate that the joystick is back to the neutral position. Comparing the *Sigma* and the *Epsilon* catheters, the clinician found that, even though the *Epsilon* actuation is more intuitive and allows one to determine the exact tip shape with the handle, the handle design could be more ergonomically convenient. While he experienced that the *Epsilon* handle allowed more choice in making S-shaped curves, he had to use both hands to make all the tip bends of choice. In addition, he experienced that the handle contacted the table surface when a too large curve downwards was made and that his arm did not have an optimal resting point.

#### Future Use

In improving the prototypes, the clinician stressed the importance of deciding whether the instruments are intended to be a catheter or a delivery sheath. While he saw potential for both options, he preferred further development towards a delivery sheath that allows a 8-8.5 Fr catheter to be placed inside. If the catheters were further developed into sheaths, the clinician envisioned their use in various procedures and would recommend them to other clinicians too. Additionally, he foresaw the catheters to be useful in all three pathway approaches: through the vena cava inferior, through the vena cava superior, and trans-aortically. The latter two are the most complex, and the added value of the *Sigma* was proven there during the passive heart setup. For the future, he would generally prefer remote control. Finally, the clinician would like to see how intact the instrument remains after prolonged and intense use in a test bed. Assuming that all improvements would be taken into account, the clinician expressed a preference for the *Epsilon* over the *Sigma*.

## Discussion

### General Findings

This study presents a first expert evaluation of a multi-steerable catheter prototype in two beating heart setups: one passively beating with pumps and a saline solution running through combined with endoscopic camera visualization and another actively beating with blood perfusion in the coronary arteries combined with echo visualization.

The passive beating heart setup proved the most suitable at this stage of catheter development and under the current circumstances to study the interaction between the catheter and the cardiac environment. The catheter successfully reached multiple locations inside the heart, including the mitral valve annulus, the left pulmonary veins, the bottom structures of the mitral valve, and the papillary muscles.

The active beating heart setup proved to be effective as a tool to study the catheter in an environment close to *in vivo* circumstances. However, during clinical circumstances, there are more possibilities to visualise the catheter than by means of the echo visualization used in the experiment. Additionally, even though a small opening was made inside the atrial septum, it was impossible to locate it using the available visualisation techniques. With no septal puncture sheath and needle being available and no modifications to the setup being allowed during active heart beating, it was impossible to cross the septum and carry out the positioning tasks. Once the catheter was introduced through an apical entrance, the clinician was able to reach the pulmonary veins, the papillary muscles, and create circular pathways at any location.

### Detailed Findings

#### Stiffness

The *Sigma* was experienced to be extremely flexible, which could lead to safer use of the instrument inside the heart, but also led to higher complexity in terms of insertion. Even though the *Epsilon* was experienced to be as flexible, the proximal curve was better able to respond to joystick manipulation than in the *Sigma* catheter.

#### Response Speed

Compared to commercially available steerable catheters, the *Sigma* scored higher on response speed. During the interview, it was confirmed that the *Sigma* was experienced significantly faster than conventional steerable catheters and that it allowed a one-to-one reaction. In addition, the *Sigma* scored higher on speed than the *Epsilon*, because, in the latter, two hands were required in some cases to create the desired S-shaped curves.

#### Workload

Following the passive beating heart experiment, the workload for the *Sigma* catheter was rated as comparable to the workload of conventional steerable catheters, except for physical demand, on which conventional catheters scored higher, and for performance, on which the *Sigma* scored poorer. Following the experiment in the active beating heart, the *Sigma* scored worse than in the passive beating heart experiment, mainly on Performance, Effort, and Frustration, likely because of experimental factors that led to challenging catheter insertion during the course of the second experiment.

#### Precision

The precision of the *Sigma* was deemed similar to that of conventional catheters, including factors such as reaching a specific location in a vessel, remaining at a specific location, and dodging a specific location. The *Sigma* catheter was more reliable in the beginning of the experiment compared to after a while, when the catheter was inside the heart, indicating a disadvantage compared to existing catheters. The *Sigma* was judged as very easy to work with for reaching each of the endpoints. The *Epsilon* was preferred regarding the precision in reaching endpoints, choosing the direction of steering, and steering along pathways.

#### Ablation Related Functionality

While both catheters were designed as multi-functional sheaths with a lumen, due to the clinician’s specialism we included a number of questions to determine the feasibility of the catheters in cardiac electrophysiology procedures. The clinician found it (very) likely that, compared to conventional catheters, the *Sigma* catheter would lead to improved manoeuvrability to reach required locations inside the heart, improved ablation along complex pathways, easier sliding of the catheter during lesion formation, easier creation of circular lesion shapes, better treatment in children, and shorter procedure time.

#### Catheter Control

The *Sigma* was rated to be ‘slightly better’ than conventional catheters when comparing the level of control over the catheter tip in the passive beating heart. In the active beating heart, however, the *Sigma* was rated to be ‘slightly worse’ than commercially available catheters on the same characteristic. These findings were in agreement with the reported workload. The clinician was positive about the use of the joysticks in the *Sigma* and found it better compared to the handles used in conventionally steerable catheters; however, he experienced difficulties as the experiment progressed. While the clinician indicated that the difficulties and his frustration were due to lacking proper visualization of the catheter and other interventional devices, the difficulties in controlling the steerability were caused as a result of blood infiltration in between the joystick components. This created friction between components that would otherwise smoothly interact with one another. Overall, the clinician preferred the *Sigma* handle over that of conventionally steerable catheters, and the *Epsilon* handle over that of the *Sigma*.

#### Intuitiveness

The clinician found both catheters more intuitive and easier to learn to work with than conventional catheters, and the *Epsilon* more intuitive than the *Sigma*, because the tip shape of the former is a direct result of the handle shape and allows one to determine the exact shape of the tip.

### Study Limitations and Future Recommendations

To compare the *Sigma* with conventional catheters, the initial idea was to physically test a conventional steerable catheter during the experiment. However, because the clinician uses multiple different ones, it was decided to not use one in the actual experiment. Instead, he stated that he could distinguish properties between the *Sigma* catheter and conventional steerable catheters as a group. Similarly, the *Epsilon* catheter was not tested in the beating heart setup but in free space, because of cables running through the outside of the prototype. Using the prototype in this early stage in the beating heart setup would have led to leakages.

Another limitation of the study is that no other typical catheterization instruments were used to simplify the catheterization procedure, such as guiding sheaths or septal puncture needles. For the future, the catheter needs to be tested with other typical catheterization instruments required for catheter insertion and withdrawal. This includes inserting the catheter *via* the femoral or upper extremity vasculature which naturally introduces greater tortuosity into the catheter pathway and overall catheter system. While the addition of this tortuosity is not expected to affect the steerability and/or responsiveness of the device,^2^ the current shaft materials may need optimization to allow sufficient pushability through the vasculature in general. The authors therefore advise follow-up experiments using an extended experimental platform that includes the vasculature.

Moreover, the current experiment focused on positioning tasks carried out by a single user which is the main limitation of this study. The aim of this study was not to repeatedly measure a specific intra-procedural endpoint such as procedure time, but rather to carry out a first user experience test to determine the instrument feasibility and steerability in a realistic cardiovascular environment and to determine design improvements prior to doing repeated measurements. When the clinician was instructed to reach a specific location with the catheter tip, the aim was to determine whether that specific steering action was possible and whether the catheter allowed doing so in a cardiac environment. Without any specific measurable endpoints at the location of interest, repeated measurements were too early to be conducted in this phase. However, repetitive experiments should be the logical next step, as well as measuring quantifiable endpoints during the experiments. Such an experiment would require changes to the catheter design and materials to allow repeated use in the cardiovascular environment and a setup that is closer to a clinical catheterization procedure. In the future, an interventional functionality, such as using the catheter together with an ablation tool, deserves to be tested by a number of clinical experts with different specialisms. In addition, because a single heart was used for each of the experiments (*N* = 2 in total), the performance of the catheter could not be tested in hearts of different sizes and with any present abnormalities. The authors advise follow-up experiments to test both prototypes in the cardiovascular environment with multiple interventional cardiologists with different specialties. To allow for a realistic test of the catheters and to quantify endpoints, the catheters should enable a specific functionality once they have arrived at the cardiac location of interest, such as cutting or ablating the tissue. Finally, the catheter should be tested in hearts with different sizes and with present abnormalities.

General design recommendations that follow from the experiment are mostly related to material optimization. Numerous (braided) shaft materials exist that could be incorporated in the design to optimize the use of the steerable catheters in the cardiovascular system without the risk of shaft kink. A robust sealing covering the tip and tip-shaft border is recommended to prevent blood from entering the internal mechanism and influencing the steerability. The tip components were designed to move along each other with rolling contact instead of sliding contact. As such, wear is negligible and no material will be lost; however, a thin-walled protective sheet or sealing would be recommended regardless. Such a sealing should not go at the cost of the steerability and requires careful selection of optimal materials for this purpose. To provide the operator positional feedback during a procedure, bands of radiopaque material are recommended to be added to the tip components to allow visibility of the steering segments under fluoroscopy. Finally, the intuitiveness of steering is an important factor in the design of any steerable catheter system. Mirrored movement between joystick and catheter tip, for example once the catheter steers in the ascending aorta, should therefore be limited. Specific future design recommendations were also made by the clinical expert for each of the prototypes. According to the expert, future design iterations of the *Sigma* should include a mechanism that provides a click response when the joysticks reach the neutral position whereas the *Epsilon* should include an improved wrist support system, allowing more space in the downward joystick location.

## Conclusion

In this study, a first expert evaluation of a multi-steerable catheter was carried out in a passive and an active beating heart experiment. The clinical expert, an electrophysiologist with over 20 years of experience, argued that the passive beating heart setup was more successful than the active beating heart setup, with insightful visualization through the endoscopic imaging while the heart was in beating condition. The steerability of the prototypes was experienced as useful and clinically relevant. Based on the questionnaires and detailed interview, we were able to identify future design improvements and developments for the steerable catheter prototypes.

## Electronic supplementary material

Below is the link to the electronic supplementary material.

Electronic supplementary material 1 (PDF 320 kb)
