# Supplementary material for: First Expert Evaluation of a New Steerable Catheter in an Isolated Beating Heart
Source: Cardiovasc Eng Technol. 2020 Nov 18;11(6):769–82. doi: 10.1007/s13239-020-00499-3 (PMC7782459; doi:10.1007/s13239-020-00499-3)
Supplement: Supplementary file 1 — Electronic supplementary material 1 (PDF 320 kb) [file 13239_2020_499_MOESM1_ESM.pdf]

1. What is your age?

____

2. What is your gender?
- ☐ Male
- ☐ Female

3. What is your dominant hand?
- ☐ Left hand
- ☐ Right hand
- ☐ Both hands

4. What is your specialism?

____

5. What is the number of years you are practising this current specialism?

____

6. Which education and training did you receive for this specialism, list them in chronological order.

____

____

7. How many hours per week are you performing electrophysiology procedures?
- ☐ Less than 10 hours
- ☐ 10 – 20 hours
- ☐ 20 – 30 hours
- ☐ 30 – 40 hours
- ☐ More than 40 hours

8. Which different types of procedures do you perform?

9. Which cardiac pathway is the most complex to move a catheter through?

10. Which cardiac location is the most complex to reach?

11. Do you have experience with steerable sheaths/catheters and if so, which ones?

12. In which cases would you typically decide to use a steerable sheath/catheter?

Thank you!

Participant: ……
Questionnaire: ……/2
Date:

Questionnaire Catheter & Cardiac Bio-simulator Experiment

Task Performance

Example: How easy was it to paint?

Very difficult 1 2 3 4 5 6 7 8 9 10 Very easy

How easy was it to manoeuvre along the pathways?

Very difficult 1 2 3 4 5 6 7 8 9 10 Very easy

How fast was it to manoeuvre along the pathways?

Very slow 1 2 3 4 5 6 7 8 9 10 Very fast

How precise was it in manoeuvring along the pathways?

Very imprecise 1 2 3 4 5 6 7 8 9 10 Very precise

How easy was it to reach the end point?

Very difficult 1 2 3 4 5 6 7 8 9 10 Very easy

How fast was it to reach the end point?

Very slow 1 2 3 4 5 6 7 8 9 10 Very fast

How precise was it in reaching the end point?

Very imprecise 1 2 3 4 5 6 7 8 9 10 Very precise

How easy was it to steer in your direction of preference?

Very difficult 1 2 3 4 5 6 7 8 9 10 Very easy

How fast was it to steer in your direction of preference?

Very slow 1 2 3 4 5 6 7 8 9 10 Very fast

How precise was it to steer in your direction of preference?

Very imprecise 1 2 3 4 5 6 7 8 9 10 Very precise

Usability

Example: I needed to learn a lot before I could paint

1	2	3	4	5
		X		

	Strongly disagree 1	2	3	4	Strongly agree 5
1. I think that I would like to use this system frequently					
2. I found the system unnecessarily complex					
3. I thought the system was easy to use					
4. I think that I would need the support of a technical person to be able to use this system					
5. I found the various functions in this system were well integrated					
6. I thought there was too much inconsistency in this system					
7. I would imagine that most people would learn to use this system very quickly					
8. I found the system very cumbersome to use					
9. I felt very confident using the system					
10. I needed to learn a lot of things before I could get going with this system					

Workload

Mental Demand — Example: How mentally demanding was it to paint?

Very Low … Very High

Mental Demand — How mentally demanding was the task?

Very Low … Very High

Physical Demand — How physically demanding was the task?

Very Low … Very High

Temporal Demand — How hurried or rushed was the pace of the task?

Very Low … Very High

Performance — How successful were you in accomplishing what you were asked to do?

Perfect … Failure

Effort — How hard did you have to work to accomplish your level of performance?

Very Low … Very High

Frustration — How insecure, discouraged, irritated, stressed, and annoyed wereyou?

Very Low … Very High

1. How would you rate the following characteristics of the multi-steerable catheter when compared to the existing steerable catheter?

	Significantly Worse	Slightly Worse	Comparable	Slightly Better	Significantly Better
**Variety in curve angles and curve types**	☐	☐	☐	☐	☐
**Level of steerability of the catheter tip**	☐	☐	☐	☐	☐
**Amount of movements that can be made with the catheter tip**	☐	☐	☐	☐	☐
**Level of control of the catheter tip**	☐	☐	☐	☐	☐
**Intuitiveness in controlling the tip**	☐	☐	☐	☐	☐
**Amount of contact force that can be applied**	☐	☐	☐	☐	☐
**Stiffness of the catheter**	☐	☐	☐	☐	☐
**Reaching a specific location in a vessel**	☐	☐	☐	☐	☐
**Reaching a specific location in a heart chamber**	☐	☐	☐	☐	☐
**Remaining at a specific location**	☐	☐	☐	☐	☐
**Maintaining a specific tip curve or angle**	☐	☐	☐	☐	☐
**Remaining instrument-tissue contact a specific location**	☐	☐	☐	☐	☐
**Sliding the catheter over tissue**	☐	☐	☐	☐	☐
**Dodging specific locations**	☐	☐	☐	☐	☐

2. How likely is it that the multi-steerable catheter will lead to the following effects?

	Very Unlikely	Unlikely	Neutral	Likely	Very Likely
**Improved manoeuvrability to reach required locations in the heart**	☐	☐	☐	☐	☐
**Improved maintenance of appropriate contact force**	☐	☐	☐	☐	☐
**Improved maintenance of appropriate rhythm control**	☐	☐	☐	☐	☐
**Improved ablation along complex pathways**	☐	☐	☐	☐	☐
**Easier sliding of the catheter during lesion formation**	☐	☐	☐	☐	☐
**Easier creation of circular lesion shapes**	☐	☐	☐	☐	☐
**Better treatment in complex anatomy**	☐	☐	☐	☐	☐
**Better treatment in infants**	☐	☐	☐	☐	☐
**Better treatment in elderly or co-morbid patients**	☐	☐	☐	☐	☐
**Shorter procedure time**	☐	☐	☐	☐	☐
**Fewer common complications**	☐	☐	☐	☐	☐
**Less tissue or blood vessel damage**	☐	☐	☐	☐	☐
**Lower recurrence rate**	☐	☐	☐	☐	☐
